# Supplementary material for: MetaRibo-Seq measures translation in microbiomes
Source: Nat Commun. 2020 Jun 29;11:3268. doi: 10.1038/s41467-020-17081-z (PMC7324362; doi:10.1038/s41467-020-17081-z)
Supplement: Supplementary file 10 — Supplementary Data 7 [file 41467_2020_17081_MOESM10_ESM.zip › File2/Confidence_High_Taxonomy/155570_out.krona.html]

Javascript must be enabled to view this page.

members
magnitude
magnitudeUnassigned
count
unassigned
taxon
rank

155570\_out

110

superkingdom
2
110

phylum
976
110

110
200643
class

110
order
171549

110
family
171550

239759
genus
110

species
1118061
110

SRS011134\_contig\_number\_23788SRS012969\_contig\_number\_40410SRS013098\_contig\_number\_44564SRS013158\_contig\_number\_1565SRS013476\_contig\_number\_36395SRS013521\_contig\_number\_2523SRS014613\_contig\_number\_11343SRS014855\_contig\_number\_11504SRS015579\_contig\_number\_2934SRS015694\_contig\_number\_2508SRS015794\_contig\_number\_1941SRS015854\_contig\_number\_contig-100\_1909.114897SRS015960\_contig\_number\_contig-100\_491.98905SRS016095\_contig\_number\_27958SRS016203\_contig\_number\_12469SRS016335\_contig\_number\_30074SRS016954\_contig\_number\_contig-100\_82.96089SRS017103\_contig\_number\_22779SRS017521\_contig\_number\_30523SRS017916\_contig\_number\_13497SRS018351\_contig\_number\_5992SRS018541\_contig\_number\_9437SRS018836\_contig\_number\_contig-100\_6568.119428SRS018936\_contig\_number\_9064SRS019381\_contig\_number\_15191SRS019445\_contig\_number\_15403SRS019496\_contig\_number\_3777SRS020328\_contig\_number\_contig-100\_1991.186315SRS020394\_contig\_number\_10972SRS021484\_contig\_number\_26019SRS021948\_contig\_number\_contig-100\_1268.202517SRS022137\_contig\_number\_8502SRS023526\_contig\_number\_22373SRS023971\_contig\_number\_contig-100\_1910.40519SRS024132\_contig\_number\_contig-100\_36506.154340SRS024625\_contig\_number\_contig-100\_4057.91977SRS043768\_contig\_number\_14611SRS045004\_contig\_number\_7025SRS045645\_contig\_number\_20050SRS045713\_contig\_number\_contig-100\_8599.93143SRS045826\_contig\_number\_12747SRS046717\_contig\_number\_8938SRS047014\_contig\_number\_contig-100\_1132.127643SRS049402\_contig\_number\_11784SRS049773\_contig\_number\_22609SRS049959\_contig\_number\_39687SRS049995\_contig\_number\_25583SRS050925\_contig\_number\_32071SRS051031\_contig\_number\_28565SRS053335\_contig\_number\_contig-100\_1807.106286SRS053398\_contig\_number\_15051SRS053649\_contig\_number\_18748SRS054352\_contig\_number\_21948SRS054905\_contig\_number\_20548SRS055017\_contig\_number\_32531SRS055982\_contig\_number\_13164SRS056259\_contig\_number\_contig-100\_11214.110940SRS056273\_contig\_number\_29938SRS057480\_contig\_number\_10308SRS064276\_contig\_number\_35131SRS065504\_contig\_number\_contig-100\_7155.160695SRS075078\_contig\_number\_24804SRS075821\_contig\_number\_15931SRS075878\_contig\_number\_12527SRS075984\_contig\_number\_20447SRS076756\_contig\_number\_13505SRS077127\_contig\_number\_636SRS077194\_contig\_number\_28448SRS077231\_contig\_number\_19642SRS077502\_contig\_number\_9351SRS078242\_contig\_number\_16967SRS097958\_contig\_number\_18654SRS098061\_contig\_number\_8958SRS098571\_contig\_number\_21461SRS098717\_contig\_number\_15901SRS098881\_contig\_number\_25257SRS101376\_contig\_number\_17606SRS101433\_contig\_number\_14782SRS1041033\_contig\_number\_16395SRS1041118\_contig\_number\_12075SRS1041132\_contig\_number\_contig-100\_2498.2499SRS1041136\_contig\_number\_2472SRS104400\_contig\_number\_27322SRS104975\_contig\_number\_7961SRS105153\_contig\_number\_4544SRS140492\_contig\_number\_26930SRS142542\_contig\_number\_17071SRS142890\_contig\_number\_19196SRS143417\_contig\_number\_9403SRS143523\_contig\_number\_476SRS143895\_contig\_number\_7947SRS143991\_contig\_number\_27373SRS144135\_contig\_number\_17763SRS144297\_contig\_number\_8877SRS144362\_contig\_number\_32531SRS144537\_contig\_number\_38437SRS145497\_contig\_number\_contig-100\_15023.53202SRS146812\_contig\_number\_61246SRS147271\_contig\_number\_contig-100\_23838.108891SRS147346\_contig\_number\_48739SRS147425\_contig\_number\_8838SRS148424\_contig\_number\_7492SRS148721\_contig\_number\_2987SRS148817\_contig\_number\_11930SRS149075\_contig\_number\_16449SRS149784\_contig\_number\_contig-100\_2520.88656SRS892882\_contig\_number\_3303SRS893369\_contig\_number\_8402SRS893383\_contig\_number\_10035SRS971275\_contig\_number\_contig-100\_395.207656
